# App2: software solution for apple leaf disease detection based on deep learning (CNN+SVM)

**DOI:** 10.3389/frai.2025.1648867

**Published:** 2025-10-15

**Authors:** Erick Aronés, Jefferson Espinal, Cesar Salas

**Affiliations:** School of Computer Science, Peruvian University of Applied Science (UPC), Lima, Peru

**Keywords:** computer vision, deep learning, CNN, SVM, apple leaf disease detection, Tensorflow, Scikit learn

## Abstract

Early detection of crop diseases is essential to reduce yield losses and improve management efficiency in agricultural production. This work presents the development of a mobile application, called App2, designed to detect diseases in apple tree leaves from images taken or uploaded by the user. The solution integrates a hybrid model based on a Convolutional Neural Network (CNN) and a Support Vector Machine (SVM), developed for computer vision tasks focused on recognizing diseases in apple leaves. The system architecture includes a user interface built with React Native, an API developed using FastAPI and deployed on Azure, and a pre-filter implemented through the OpenAI API to validate that the uploaded images correspond to crop leaves. The model was trained to classify images into six categories: Scab, Black Rot, Rust, Healthy, Powdery Mildew, and Spider Mite. Experimental results showed a 95% success rate in test cases and 80% performance in detecting clear images of affected leaves. User evaluations indicated high usability and satisfaction, demonstrating that the mobile application has strong potential as an accessible and effective technological tool for disease monitoring in apple crops.

## 1 Introduction

Apple production plays a fundamental role in Peru’s agricultural economy, with more than 11,000 hectares under cultivation, and the Lima region concentrates for approximately 80% of national production (Redacción Agraria, 2016; Arias, 2024). In 2023 alone, and considering the growth projection based on irrigation projects, this activity generated revenues exceeding 85 million soles, establishing itself as one of the most profitable crops in the country (Ministerio de Desarrollo Agrario y Riego del Perú, n.d.). However, its productivity is constantly threatened by foliar diseases caused by fungi and bacteria, whose incidence is worsened by poor agricultural practices and unpredictable climatic phenomena such as El Niño (Melo, 2023). In southeastern areas of Lima, these diseases have reduced production by up to 20% due to a lack of phytosanitary knowledge and the absence of appropriate treatments (Programa Nacional de Innovación Agraria, 2020). This situation makes early and accurate diagnosis indispensable. However, traditional disease identification is complex due to the visual similarity of symptoms among different pathogens, as well as the influence of environmental variables such as humidity, light and temperature. Added to this is the reliance on human judgment and the limited accessibility to remote agricultural areas, which hinders timely intervention. Although deep learning-based solutions exist globally, many current models are too complex or demanding to be effectively implemented on mobile devices.

In this context, App2, a deep learning-based hybrid model combining convolutional neural networks (CNN) for feature extraction and support vector machines (SVM) for classification, is proposed. As a result, it achieved 95% accuracy in detecting six main conditions in apple tree leaves: Black Rot, Rust, Scab, Healthy, Powdery Mildew and Spider Mite. Its lightweight architecture, with only 98.4 K parameters, makes it ideal for integration into a mobile application that performs detection in real time, with processing in the cloud. This solution is framed in the field of computer vision, applying advanced image analysis techniques to identify characteristic visual patterns of diseases and pests. This tool aims to provide farmers with an accessible, reliable and fast solution that enables timely decisions to be made in the field. By providing accurate detection without the need for expensive equipment or advanced technical expertise.

## 2 Review literature

In recent years, several approaches based on machine learning and computer vision have been proposed to detect diseases in crops, using image analysis techniques to detect visual symptoms automatically and accurately. Bi et al. (2020) developed an apple leaf disease identification system using the MobileNet architecture, a lightweight convolutional neural network model designed to operate efficiently on mobile devices. To achieve good performance, the model was trained on a dataset collected by agricultural experts in Shaanxi, China, including images of leaves affected by Alternaria leaf blotch and rust. Data augmentation techniques such as rotation, cropping and grayscale scaling were applied, increasing the robustness of the model to image variations. Experimental results showed that MobileNet achieves 73.50% accuracy with an average processing time per image of 0.22 s, which is significantly faster than models such as InceptionV3 (75.59% in 0.45 s) and ResNet152 (77.65% in 0.79 s). Although ResNet152 achieved slightly higher accuracy, its efficiency is much lower, making it less viable for mobile applications. Finally, the limited dataset used (2,004 images) affects the system’s accuracy and generalization.

Sanida et al. (2022) proposed a hybrid model based on convolutional neural networks (CNN) for accurate tomato leaf disease detection using images from the PlantVillage dataset. The architecture combines blocks of the VGG16 model with an Inception module, thus leveraging the ability of both to extract features at multiple scales and depths. The model was trained with over 76,000 images generated using data augmentation techniques such as rotations, translations, scaling and mirroring. This hybrid structure includes 13 convolutional layers, five max-pooling layers, an Inception module, a global average pooling layer and a softmax classification layer, all optimized with the Adam algorithm and a learning rate of 0.0001. The results of the hybrid model outperformed advanced models such as ResNet152 and InceptionResNet, achieving an accuracy of 99.17%, recall of 99.23%, F1-score of 99.17% and an AUC of 99.56%. This hybrid approach proves to be highly effective for agricultural applications. However, a potential limitation is that the dataset used consisted of images taken under controlled conditions, with neutral backgrounds and centered leaves, which may restrict the model’s generalization in real field scenarios where illumination and background variability are significant factors.

Ksibi et al. (2022) proposed MobiRes-Net, a hybrid deep learning model integrating ResNet50 and MobileNet architectures, designed to detect and classify olive leaf diseases. A total of 5,400 leaf images were collected using a drone in the Al Jouf region of Saudi Arabia and classified into four categories: healthy leaves, *Aculus olearius*, olive scab, and peacock spot. The images were preprocessed (resized to 224 × 224 pixels, normalized and contrast enhanced) and subjected to data augmentation techniques to improve model robustness and avoid overfitting. MobiRes-Net was trained with pre-trained weights, replacing the original classification layers of each network and joining their fully connected layers to form a joint classifier with Softmax at the output. Implemented with TensorFlow and Keras, the model achieved a classification accuracy of 97.08%, significantly outperforming ResNet50 (94.86%) and MobileNet (95.63%). In addition, it obtained an F1-score of 96.86% and recall of 97.11%, showing consistent results in all classes. However, despite its high accuracy, the complex structure of the hybrid model results in longer training and inference times compared to individual architectures, and it requires high-performance hardware.

Nag et al. (2023) presented a mobile application for tomato leaf disease identification using convolutional neural networks (CNN) and transfer learning techniques. Five models pre-trained on ImageNet (AlexNet, ResNet-50, SqueezeNet-1.1, VGG19, and DenseNet-121) were fine-tuned to classify ten tomato disease classes, including healthy images. The dataset included more than 19,000 images, sourced from the PlantVillage repository and a proprietary database captured in the field. Data augmentation transformations (rotation, zoom, flip) were applied to improve generalization. The DenseNet-121 model was the top performer, achieving 99.85% accuracy, outperforming VGG19 (99.74%) and ResNet-50 (99.79%). Training was conducted for 40 epochs using the Adam optimizer and fine-tuning of the last layers to fit the 10 classes. As a result, a cross-platform mobile app was developed, built with Flutter and a Django backend, which allows users to take or upload a photo of a tomato leaf, send it to a web server for processing, and receive predictions within seconds. The app, available in English, Hindi, and Assamese, was designed to promote accessibility among Indian farmers with limited English proficiency. While the system demonstrates high accuracy and usability, its performance remains highly dependent on the quality of input images. Low resolution, poor lighting conditions, or blurred captures from smartphone cameras can introduce noise or bias, potentially leading to misclassifications in real-world use.

Chen et al. (2024) proposed a lightweight method for detecting grapevine leaf diseases using an improved version of YOLOv8, called YOLOv8-ACCW, aimed at increasing detection accuracy and facilitating deployment on mobile devices. The model targeted three common grapevine diseases—black root, black measles, and blight—and integrated several modules to overcome limitations of previous algorithms. These included the AKConv module, which replaces traditional convolutions to enable arbitrary sampling and reduce parameters; the Coordinate Attention (CA) mechanism, which enhances feature extraction while suppressing irrelevant information; and the CARAFE module, which improves feature reassembly capabilities. In addition, the traditional loss function was replaced with Wise-IoU to optimize bounding box regression, particularly in small disease regions. The model achieved an F1-score of 92.4%, mAP50 of 92.8%, and mAP50-95 of 73.8%, outperforming the original algorithm in both accuracy and computational efficiency. Despite these advances, the study highlights the need to expand the dataset to include a broader variety of grapevine diseases and to refine the algorithm’s robustness under challenging real-world conditions such as strong reflection or extremely low-light environments.

Pineda Medina et al. (2024) presented the development of a lightweight offline mobile application for disease detection in potato crops. Using images from the PlantVillage dataset, the authors evaluated five CNN architectures (MobileNetv2, VGG16, VGG19, InceptionV3, and Xception), with MobileNetv2 standing out for its balance between accuracy (98.7%) and computational efficiency. The application, compatible with Android 4.1 and above and not requiring an internet connection, was designed to detect common potato diseases such as early blight and late blight from leaf images captured in real time or selected from the gallery. The MobileNetv2 model was trained in three stages with a progressive increase in layers and epochs, achieving high accuracy without overfitting. Compared to more complex architectures, it proved to be the most suitable for direct integration into mobile devices due to its small size (3.89 MB) and low number of trainable parameters. Furthermore, the application includes an informative section on potato diseases. As a limitation, the authors highlight the need to expand the system to classify additional pathologies such as rhizottoniosis and bacteriosis, which are also relevant in potato cultivation.

## 3 Materials and methods

### 3.1 Dataset

For the training of the proposed model, a dataset composed of apple leaf images classified according to the disease present was used. All images were resized to a uniform size of 256 × 256 pixels and normalized by dividing the pixel values by 255. The construction of the dataset followed a balancing strategy: all available images from the PlantVillage dataset were included (22.6% of the total), and complemented with additional images from Plant Pathology 2021 until reaching 2,000 images per disease class (Scab, Black Rot, Rust) and 4,000 images for the Healthy class. This ensured both representativeness of controlled laboratory conditions and incorporation of visual variability from field environments (e.g., heterogeneous lighting, natural backgrounds, overlapping leaves), which accounted for approximately 65.6% of the final dataset.

Two additional classes of high relevance for Peruvian agriculture were incorporated: Spider Mite and Powdery Mildew (Servicio Nacional de Sanidad Agraria del Perú, 2020). Images of Spider Mite were obtained from the ARoja 2022 dataset available in Mendeley Data, while Powdery Mildew images were sourced from the Apple Leaf Disease Powdery Mildew dataset on Kaggle. Both sources contain field-collected, labeled images suitable for model training.

To increase intra-class variability and achieve dataset balance, data augmentation was applied using Keras’ ImageDataGenerator with the following parameters: random rotation up to 40°, horizontal and vertical translations up to 20% of the image size, horizontal and vertical flips (probability = 0.5), zoom range up to 20%, and nearest-neighbor filling for missing pixels. These transformations generated approximately 1,653 additional synthetic images (11.8% of the dataset), resulting in a final set of 14,000 images distributed across six balanced classes (see Table 1).

**Table 1:** Number of images per class.

Class	Plant village	Plant pathology 2021	Apple leaf disease powdery mildew	ARoja 2022	Data augmentation	Total
Scab	630	1,370	–	–	–	2,000
Black Rot	621	1,379	–	–	–	2,000
Rust	275	1,725	–	–	–	2,000
Healthy	1,645	2,355	–	–	–	4,000
Spider Mite	–	–	–	1,163	837	2,000
Powdery Mildew	–	–	1,184	–	816	2,000
Total	3,171	6,829	1,184	1,163	1,653	14,000

**Figure 1:**
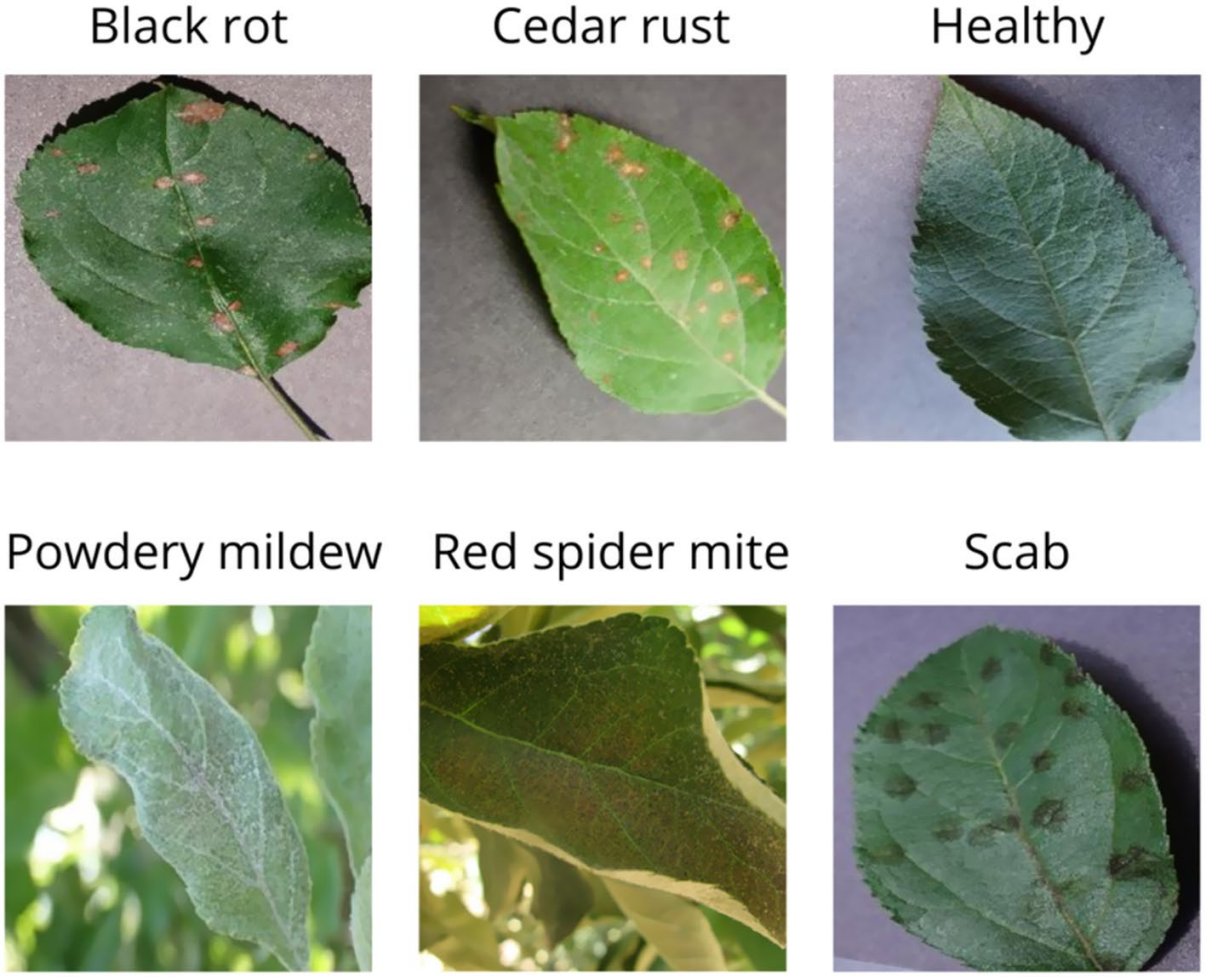
Dataset classes for App2 model training.

This strategy allowed the construction of a balanced and visually diverse dataset, reflecting both laboratory and real-world scenarios. The inclusion of field images with varied conditions strengthens the generalization ability of the model when used in mobile devices for practical applications. Figure 1 shows the classes contained in our training dataset.

### 3.2 Proposed CNN+SVM model

In recent years, the combination of Convolutional Neural Networks (CNNs) with Support Vector Machine (SVM)-based classifiers has gained popularity in various studies related to computer vision and image classification, particularly in biomedical and agricultural applications (Jia et al., 2020; Vijayalakshmi, 2020; Deepak and Ameer, 2021; Peng et al., 2021).

**Figure 2:**
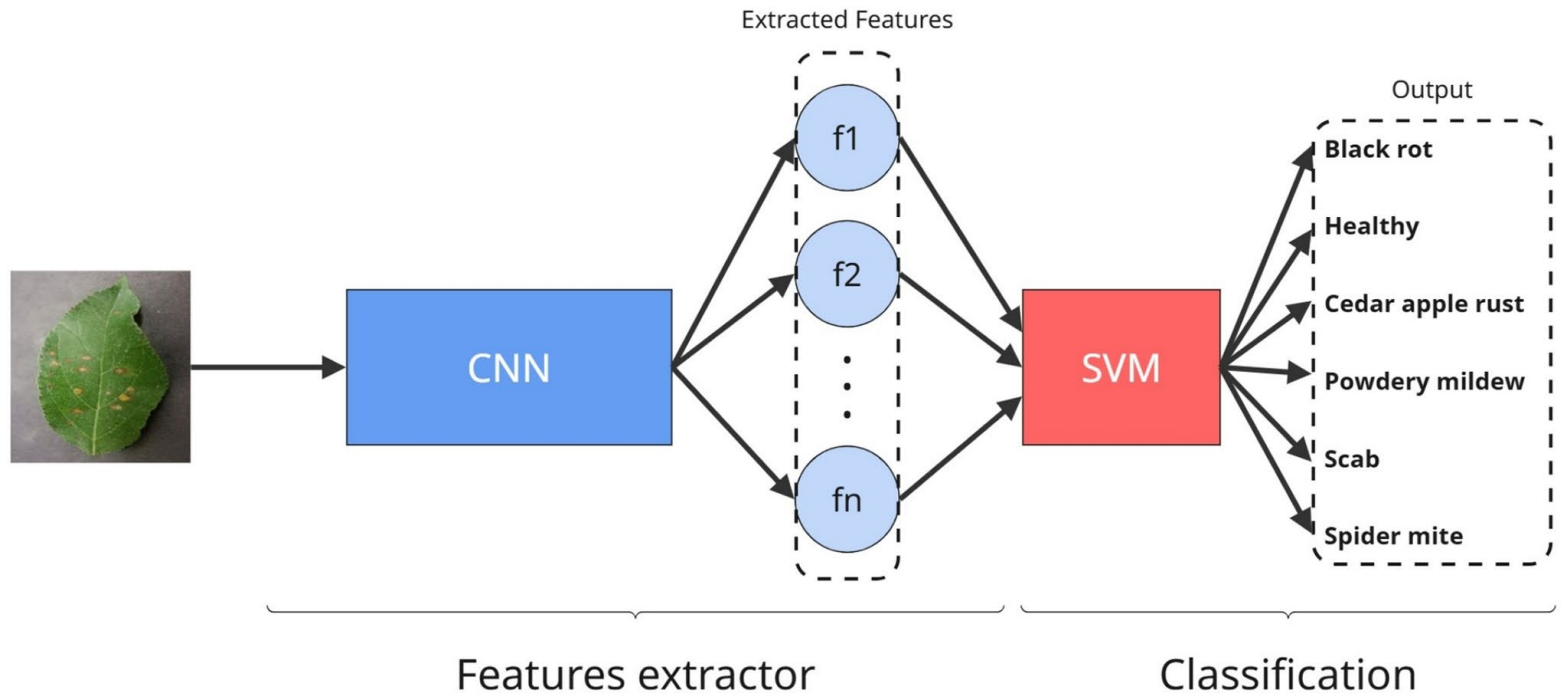
Architecture of the proposed CNN+SVM model.

As shown in Figure 2, this hybrid architecture is based on the ability of CNNs to automatically and hierarchically extract discriminative features from complex visual data, and on the strength of SVMs as robust classifiers capable of finding optimal boundaries in high-dimensional spaces. Numerous studies have shown that replacing traditional dense layers with an SVM classifier in the final stage improves model generalization, reduces overfitting, and yields higher accuracy on limited or imbalanced datasets (Khairandish et al., 2022; Surono et al., 2023).

**Figure 3:**
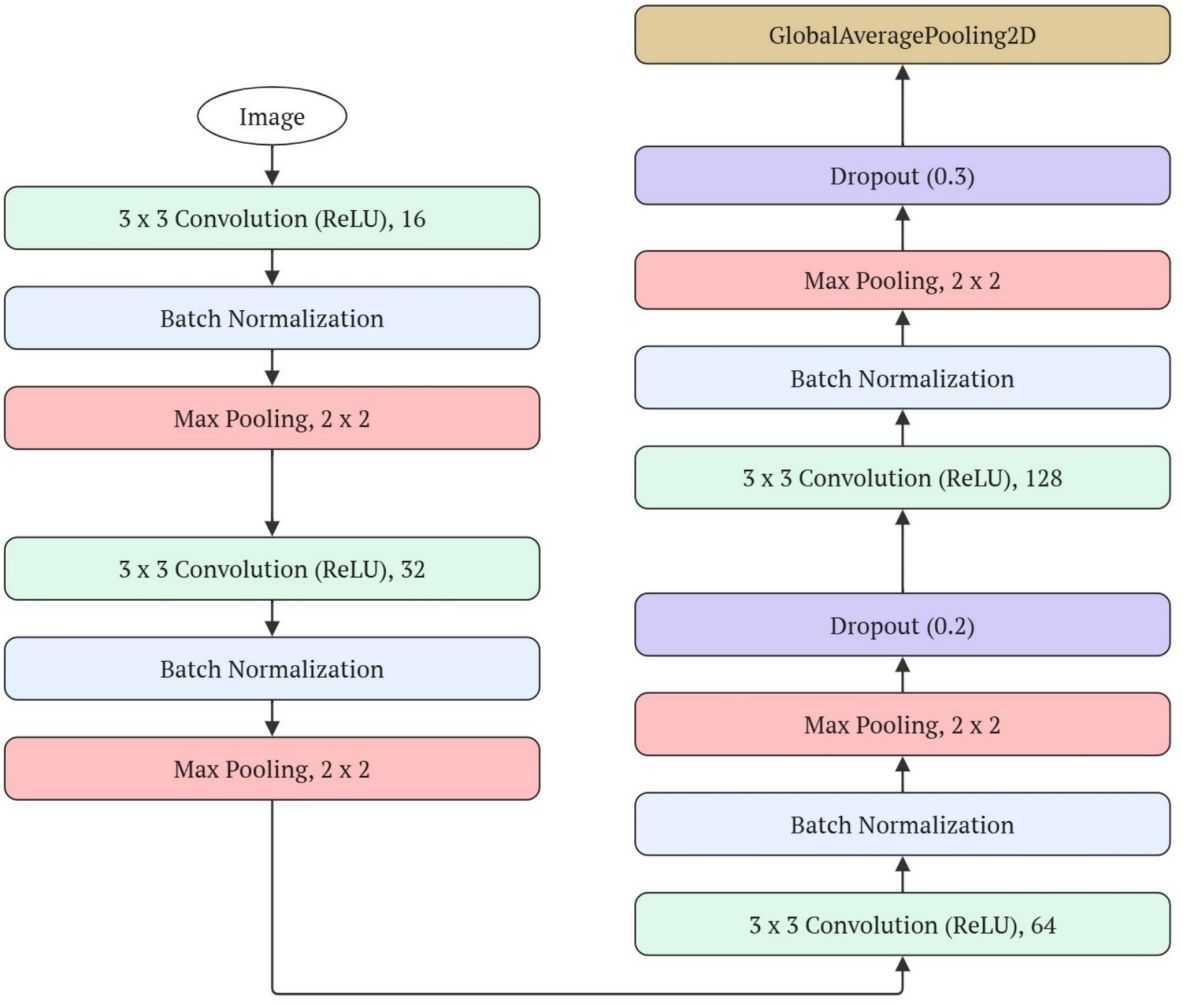
The flow of the CNN feature extractor of the proposed model.

The proposed model’s process begins with the acquisition of apple leaf images, which are resized to a uniform resolution of 256 × 256 pixels to ensure consistency in processing. As shown in Figure 3, these images are fed into a Convolutional Neural Network (CNN) composed of multiple blocks structured with convolutional layers, ReLU activation, Batch Normalization, and Max Pooling (Ioffe and Szegedy, 2015; Galanis et al., 2022). Through this architecture, the network is able to extract important visual features such as edges, textures, spot patterns, and color variations that are indicative of diseases like Scab, Black Rot, Rust, Powdery Mildew, and Spider Mite (Alzubaidi et al., 2021). As the data moves deeper into the network, the number of filters increases (32, 64, 128, and 256), allowing the model to capture both low- and high-level information. Dropout layers with a rate of 0.3 are also integrated to mitigate overfitting and enhance the model’s generalization capacity (Schmidhuber, 2015).

After processing through the CNN, a Global Average Pooling 2D layer is applied, transforming each activation map into an average value and generating a compact and discriminative feature vector (Zhou et al., 2016). This vector is used as input for a Support Vector Machine (SVM) classifier, which projects the data into a high-dimensional space to find a hyperplane that optimally separates the classes: Black Rot, Healthy, Powdery Mildew, Rust, Scab, and Spider Mite (Chandra and Bedi, 2021). During the training phase, the CNN learns to identify visual patterns, while the SVM adjusts the parameters that define the boundaries between classes.

**Figure 4:**
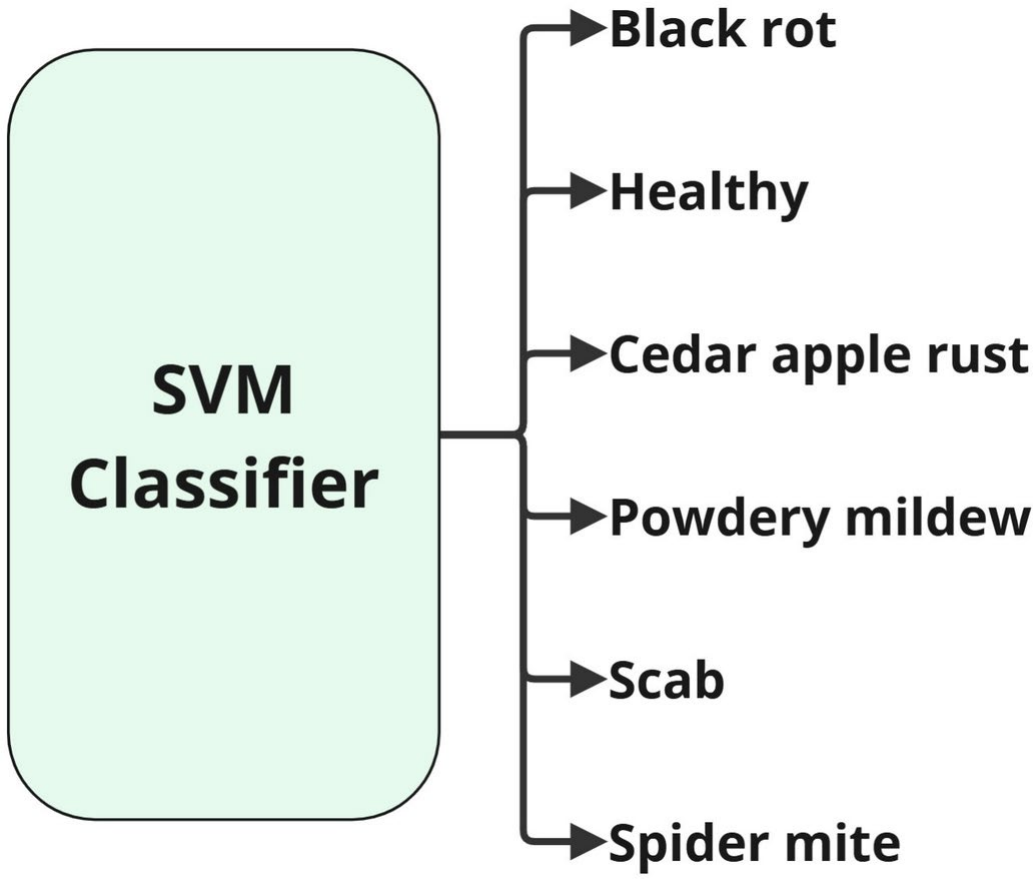
Diagram of the classifier of the proposed model.

In the inference stage, a new image is processed by the CNN to extract its features, which are then classified by the SVM, accurately determining the presence of a disease or confirming the health of the leaf. Figure 4 illustrates the SVM algorithm diagram.

### 3.3 Model training and configuration

**Figure 5:**
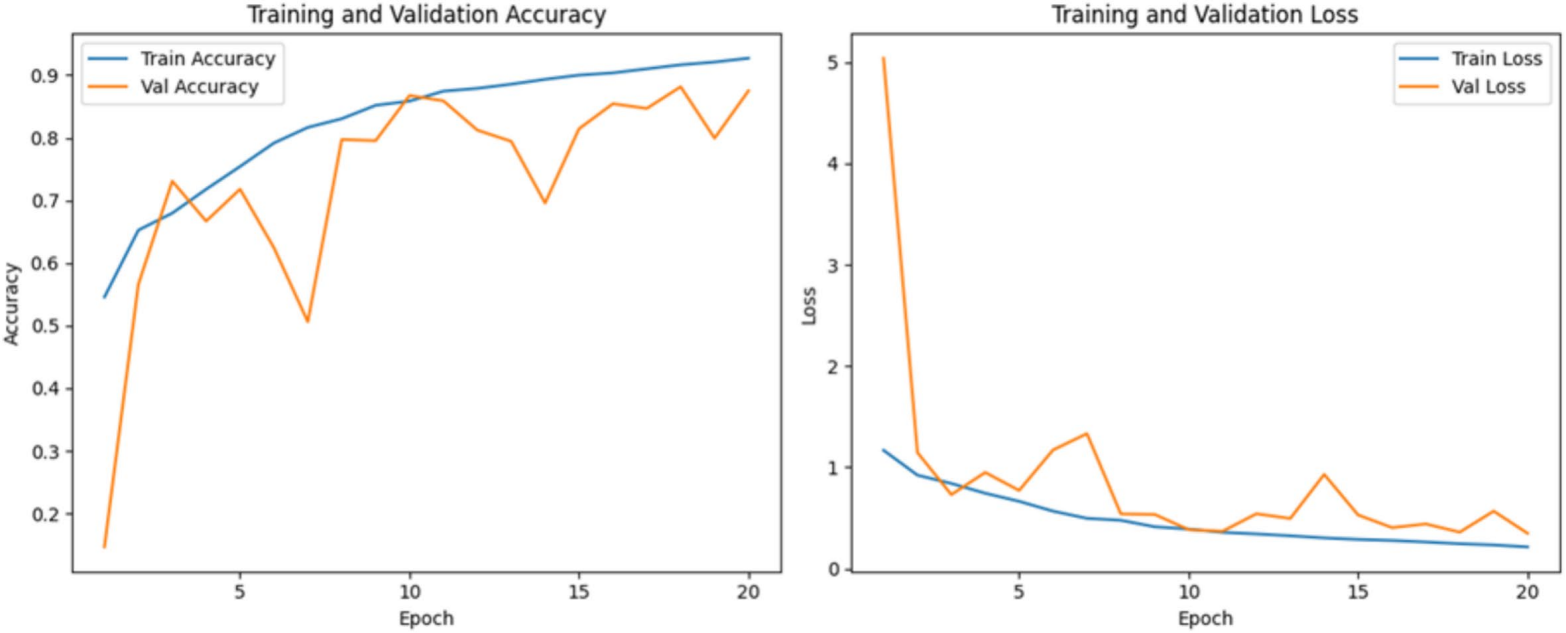
Accuracy and loss curves during CNN training.

The convolutional neural network (CNN) was trained using the Adam optimizer with a constant learning rate of 0.001. The model was trained for 20 epochs with a batch size of 32. The training and validation curves of accuracy and loss are presented in Figure 5. The training accuracy shows a consistent upward trend across the 20 epochs, while the validation accuracy fluctuates, stabilizing above 85% in the later epochs. This behavior suggests that the model is learning effectively, although some variance in validation results may indicate sensitivity to specific samples. The training and validation loss curves further support this conclusion: training loss decreases steadily, and although the validation loss exhibits some oscillation, it generally remains low, indicating good generalization and no significant overfitting.

After training, the feature vectors extracted from the Global Average Pooling layer of the CNN were used as input to a support vector machine (SVM) classifier. A grid search strategy was employed to optimize the SVM hyperparameters. The regularization parameter C was tested with values [0.1, 1, 10], and two kernel types were evaluated: linear and radial basis function (rbf). The best result was obtained using C = 10 and an RBF kernel, achieving a mean test score of 95.67%, as shown in Table 2.

**Table 2:** Results of SVM hyperparameter tuning using GridSearchCV.

C	Kernel	Mean test score
10	rbf	95.67%
1	rbf	94.85%
0.1	linear	94.63%
1	linear	94.57%
10	linear	93.71%

### 3.4 App2 mobile application

The mobile application was developed using React Native, a cross-platform development framework that allows creating native Android mobile applications from a single code base in TypeScript (Biørn-Hansen et al., 2019). This technology facilitated an agile and adaptable implementation to different devices (Liu et al., 2025). The user interface was designed to be intuitive and accessible, allowing the user to perform the following main actions:

- Take a picture directly from the device camera. (Android 6.0 as minimum version)
- Upload an image from the phone’s photo gallery.
- Send the image to the server for analysis using an API.
- Display the detection of the model, along with a brief description of the detected disease and basic recommendations.

**Figure 6:**
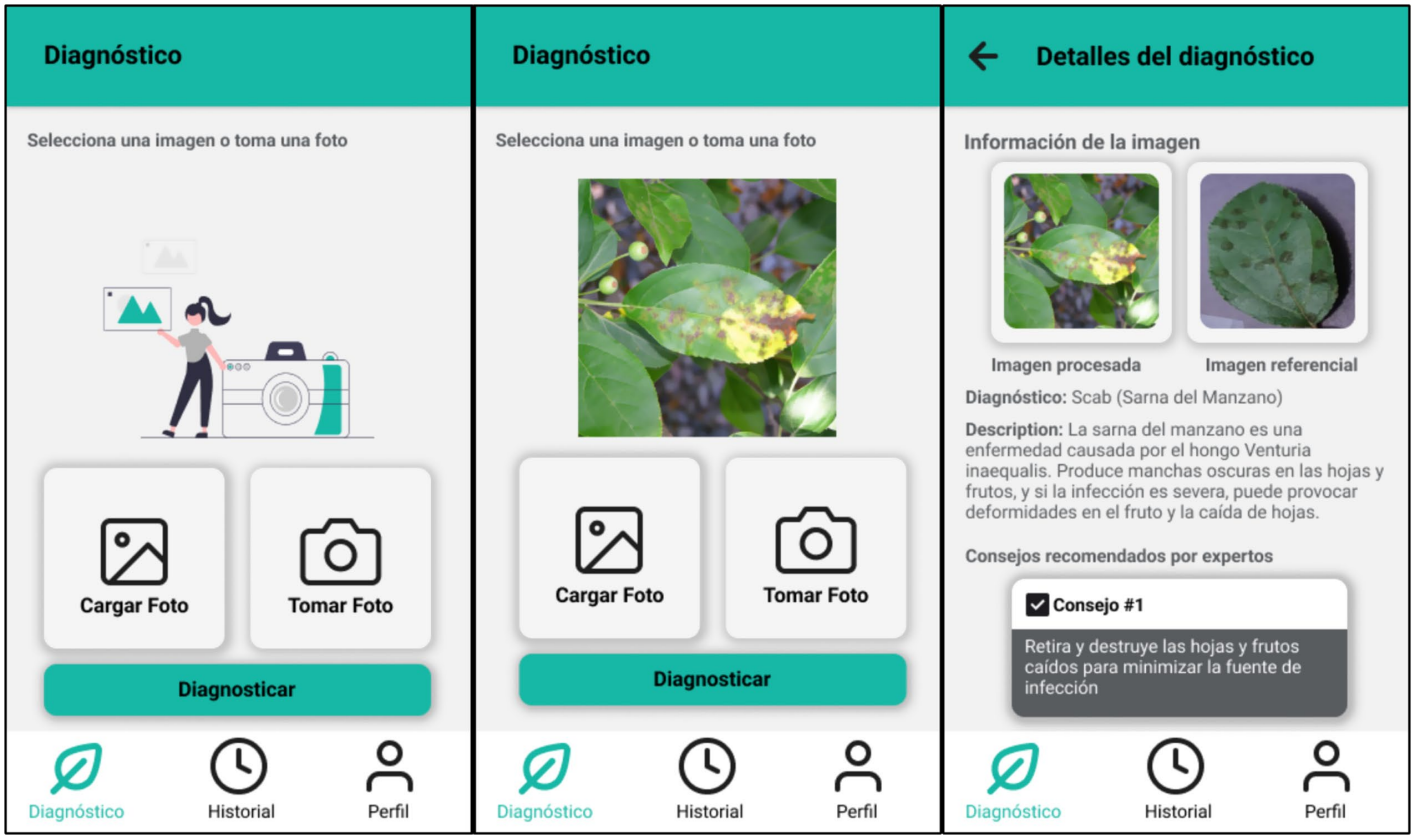
Interfaces of the mobile application detection process.

Clean and functional visual components were implemented, ensuring a smooth user experience as can be seen in Figure 6.

### 3.5 Integration of the model with the mobile application

The detection model and the mobile application are connected in an organized manner, with each part fulfilling a clear function within the solution architecture. First, the application is accessed from an Android mobile device with an internet connection. Through this app, the user can take a photograph or upload an existing image of an apple tree leaf with the goal of immediate detection.

The mobile interface was developed using React Native, enabling a seamless user experience (Komperla et al., 2022). Once the user selects or captures an image, it is sent to the server via an API developed with FastAPI and Python. This API is hosted on the Azure cloud platform, ensuring the scalability and availability of the service (Al-Sayyed et al., 2019; Gundu et al., 2020). For storing information related to diagnostics, user logs, and other interactions, an SQLite database is used, chosen for its lightweight nature and ease of integration with FastAPI-based services (Jeon et al., 2012; Gaffney et al., 2022).

Before the image is analyzed by the detection model, an intermediate validation step is executed using the OpenAI API. This filter is responsible for confirming whether the image submitted by the user indeed contains a crop leaf. If the image does not meet this criterion, the system notifies the user and prompts them to upload or capture a different photo. This step is essential to prevent erroneous detections and maintain the system’s quality, as it ensures that only valid images are processed.

**Figure 7:**
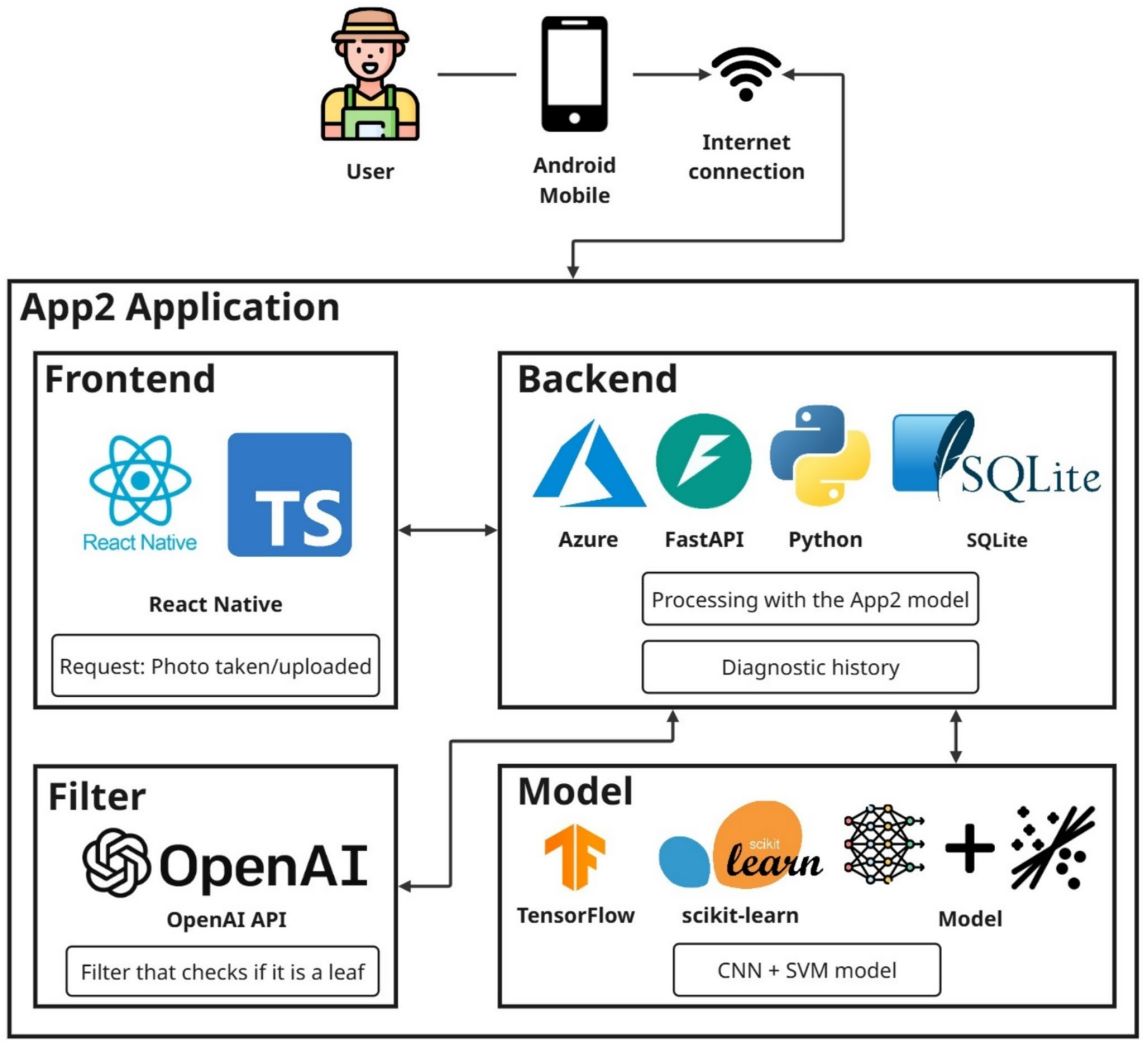
App2 mobile application architecture diagram.

If the image passes the validation filter, it is sent to the detection model, which consists of a hybrid CNN+SVM architecture. The Convolutional Neural Network (CNN) is developed using TensorFlow, while the Support Vector Machines (SVM) component is implemented with the scikit-learn library (Pedregosa et al., 2011; Hao and Ho, 2019; Pang et al., 2020). Once the disease detection process is completed, the results are returned to the user via the mobile application. This end-to-end flow delivers a robust and reliable user experience by coherently and efficiently integrating artificial intelligence technologies with mobile development. Figure 7 illustrates the architecture diagram of the App2 mobile application.

## 4 Experiments

### 4.1 Experimental protocol

A comparative evaluation was conducted using three widely adopted lightweight CNN architectures: MobileNetV2, EfficientNetB0, and ResNet50. The assessment employed consistent metrics, including accuracy, precision, recall, and F1-score, to objectively compare the classification performance of each model. Additionally, an ablation study was carried out to examine the impact of the OpenAI-based image validation filter on the overall system performance. For this purpose, the same dataset was used to evaluate two configurations of the diagnosis system: one incorporating the validation filter to ensure the uploaded image contains a leaf, and another without it. This comparison aimed to determine the filter’s contribution to reducing misclassifications and improving the reliability of the diagnostic output.

**Figure 8:**
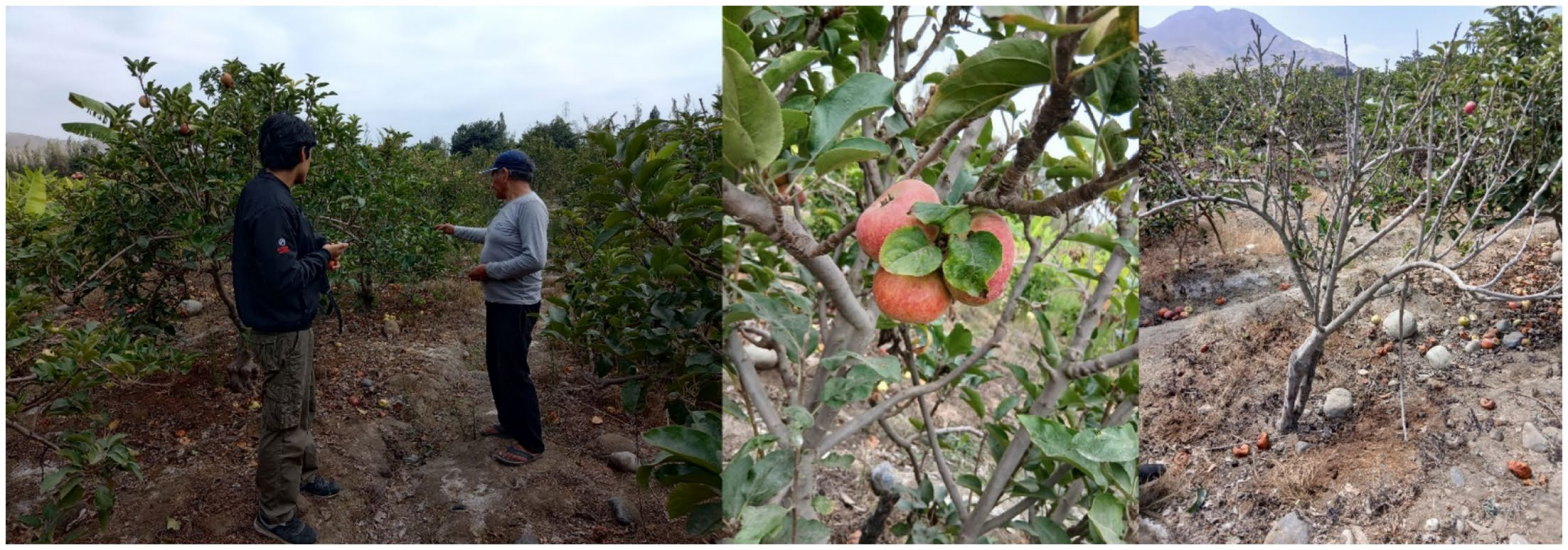
Photos of the validation process in Huaura (Lima—Peru).

The experimental evaluation of the App2 mobile application follows a rigorous and systematic approach, aimed at validating both the technical performance of the classification model and the user experience. The main objective of this evaluation is to ensure that the tool is accurate, functional, and practical in real-world agricultural scenarios. Figure 8 shows the photos of the validation process.

The experimental protocol is designed to confirm that the machine learning model integrated into the application can effectively detect various diseases from images captured by users themselves, while maintaining a simple and intuitive user experience.

In addition to technical tests focused on the model’s accuracy and the API’s responsiveness, a user experience evaluation component has also been included. For this purpose, a questionnaire will be applied, containing statements regarding ease of use, interface clarity, detection speed, and the perceived usefulness of the results provided by the application.

The experimental protocol is structured around two main components:

- Technical performance testing: A set of test cases will be applied to evaluate key functionalities of the application, such as image upload and capture, data transmission to the server, receipt of detection results, and proper visualization of results in the interface.
- User experience questionnaire: Agricultural sector users will participate in a field test, after which they will complete a questionnaire designed to assess aspects such as ease of navigation, clarity of results, and overall satisfaction.

This dual approach provides a comprehensive view of both the technical performance of the application and its perceived acceptance and usefulness by users. Specific details of the protocol, including test cases and questionnaire content, are presented in the Appendix.

### 4.2 Results

To benchmark the performance of the proposed hybrid model, a comparative analysis was conducted using well-known convolutional neural network (CNN) architectures. These models are widely recognized for their efficiency and accuracy in image classification tasks. All models were trained and evaluated on the same dataset, and performance was assessed using four key metrics: accuracy, precision, recall, and F1-score, ensuring a consistent and objective comparison across all approaches. All CNN architectures were trained using the Adam optimizer with a constant learning rate of 0.001. Each model was trained for 20 epochs with a batch size of 32, ensuring stable convergence while maintaining computational efficiency.

The choice of a hybrid CNN+SVM architecture over standalone CNN models was based on the justification provided in Section 3.2 and reinforced by our experimental results. In comparative tests, CNN+SVM consistently achieved higher accuracy and more stable results than CNNs trained with a softmax classifier, such as MobileNetV2, EfficientNetB0, and ResNet50, when evaluated on the same dataset. This improvement highlights the SVM’s ability to reduce overfitting and enhance generalization, especially in heterogeneous datasets with varying image conditions. Additionally, the lightweight CNN backbone with only 98.4 K parameters ensures efficient integration into mobile devices, while the SVM layer strengthens robustness without significantly increasing computational cost. This makes the hybrid approach more suitable for real-world mobile applications in agriculture compared to individual CNN architectures. The results, summarized in Table 3, demonstrate the effectiveness of the proposed hybrid model relative to other lightweight CNN baselines.

**Table 3:** Comparison of the App2 model with traditional CNN models.

Model	Parameters	Accuracy	Precision	Recall	F1-score
MobileNetV2	3.4 M	89.00%	88.97%	89.00%	88.92%
EfficientNet0	5.3 M	91.20%	91.00%	91.10%	91.05%
Xception	22 M	84.08%	84.05%	84.08%	84.04%
ResNet50	25.6 M	92.30%	92.15%	92.25%	92.20%
App2 model	98.4 K	94.91%	95.24%	94.65%	94.93%

To assess the effectiveness of the OpenAI API-based filter, we tested its performance on a set of 500 manually labeled apple leaf images. The filter is designed to reject images that do not contain leaves before they are sent to the diagnostic model. The prompt guides the model to evaluate the visibility and clarity of leaves in the image. Below is the exact prompt used:

System prompt: “You are an image screening system specialized in detecting apple leaves. Your task is to determine whether the image contains a clearly visible apple leaf. The leaf must be well-focused, distinguishable from the background, and well-lit. Ignore images that are blurry, too dark, occluded, or where the leaf cannot be clearly identified. Only return ‘1’ if you are confident that a healthy or diseased leaf is clearly visible; otherwise, return ‘0’.”

The results are summarized in Table 4.

**Table 4:** Performance of the leaf validation filter.

Filter result	Number of images	Percentage
Accepted (leaf)	482	96.4%
Rejected (non-leaf)	18	3.6%
Total	500	100%

The filter correctly accepted 96.4% of valid images, demonstrating high accuracy and minimal loss of relevant data. Its inclusion helps reduce false positives, increases diagnostic precision, and streamlines the overall image pipeline by ensuring only clean, relevant content reaches the disease classification model.

The tests, which covered various cases, were conducted to evaluate the performance of the App2 mobile application. The success rates of these tests are presented in Table 5.

**Table 5:** Success rate of each test case.

Test case	Description	Success rate
001	Taking a photo for analysis	70%
002	Loading a saved photo	80%
003	Successful detection with clear image	80%
004	Display model result	100%
005	Diagnostic recommendations	80%
006	Successful access to history	100%
007	Deleting a diagnostic history record	100%
008	Cancel deletion of a diagnostic history record	100%
009	Successful registration with basic data	100%
010	Weak or mismatched password	100%
011	Successful login	100%
012	Failed login	100%
013	Update user data	100%
014	Account deletion	100%
015	Cancel account deletion	100%
016	Update notification	100%
017	Show guidance	100%

The overall success rate was 95%, which reflects a high level of technical performance, with some cases showing areas for improvement mainly related to image capture.

In Case Test 001, the user was required to enter the “Diagnosis” section, capture an image of a leaf through the device’s camera, and confirm the image for processing. The test obtained a 70% success rate, which, although not reaching full effectiveness, demonstrated that the system was able to process most of the captured images correctly. However, errors were mainly associated with external factors such as variable lighting (e.g., shadows or strong sunlight), limitations in device camera resolution, and suboptimal capture angles. To address these issues, future work should explore improvements such as recommendations for standardized image capture angles and distances to guide the user before submitting an image. These improvements would strengthen the reliability of the system in real-world field conditions.

The improvement observed in Test Case 003, which increased from 40 to 80%, can be explained by the combination of algorithmic enhancement and operational optimization. On the algorithmic side, the hybrid CNN+SVM model was strengthened through the expansion of the dataset to 14,000 images, of which 65.6% corresponded to field conditions. This expansion, together with the inclusion of two additional classes of high local relevance (Powdery Mildew and Spider Mite), allowed the model to achieve an intrinsic classification accuracy of 94.91%, providing the necessary foundation for reliable detection.

From an operational perspective, the integration of the OpenAI-based pre-filter was decisive in mitigating failures during real-world use. With an effectiveness of 96.4% in validating leaf presence, this filter prevented irrelevant or low-quality images (e.g., hands, soil, blurred backgrounds) from entering the system, ensuring that the high accuracy of the model was effectively translated into application performance.

In parallel to the functional tests, a satisfaction questionnaire was applied to users who used the application in real conditions. The statements were rated on a scale from 1 (very dissatisfied) to 5 (very satisfied). The results are presented in Table 6.

**Table 6:** Questionnaire results.

Statement	Likert scale: 1 (very dissatisfied) to 5 (very satisfied)				
	1	2	3	4	5
I think I would like to use this app frequently to detect diseases on apple tree leaves.	0%	0%	20%	20%	60%
I found the application easy to use.	0%	0%	0%	20%	80%
I think I would need technical help to be able to use this app.	0%	0%	40%	60%	0%
The app is well organized and everything makes sense to each other.	0%	0%	0%	0%	100%
I did notice that sometimes the app would not work the same or would crash.	100%	0%	0%	0%	0%
Learning to use this app was quick and easy for me.	0%	0%	0%	80%	20%
I found the app difficult or confusing to use.	100%	0%	0%	0%	0%
I needed to learn many things before I could use the application correctly.	0%	0%	0%	100%	0%
The results provided by the model matched your expectations or knowledge about leaf disease.	0%	0%	0%	80%	20%
The app detected the disease in a reasonable time.	0%	0%	0%	20%	80%

The user experience evaluation was conducted with five farmers from the Huaura district (Lima, Peru), all of whom actively work in apple cultivation. The participants were male, aged between 36 and 68 years, with agricultural experience ranging from 10 to more than 30 years. Their educational background varied from primary to secondary school, and while their formal technical training was limited, they possessed sufficient practical familiarity with smartphones to operate mid-range Android devices. Two support users assisted in the process but did not participate in the questionnaire.

To assess usability, a questionnaire with a scale of 1 to 5 was applied to measure the participants’ perception of the usability, organization and intention to use the application. The responses reflected a high level of acceptance and overall satisfaction. For example, 60% of users strongly agreed that they would use the application frequently to detect diseases in apple crops, and an additional 20% agreed, demonstrating a clear willingness to use the tool repeatedly.

Ease of use was one of the most highly rated aspects. 80% of the participants found the application easy to use (value 5) and the remaining 20% also agreed (value 4), indicating unanimity in terms of the navigation experience. In addition, 100% of users strongly disagreed that the application was confusing or difficult to use. This perception is reinforced by the statement “Learning to use this application was quick and easy,” where 100% gave positive values (80% at level 4 and 20% at level 5).

Regarding the visual and structural organization of the app, the results were equally positive: 100% of users strongly agreed that the app was well organized and that everything made sense. In terms of technical stability, all participants reported experiencing no crashes or inconsistent behavior during use, which is a strong indicator of the system’s functional robustness.

An interesting point was the perception regarding the need for technical support or prior learning. Although 60% of users agreed that they needed to learn several things before using the application properly, these same participants rated the ease and speed of learning positively. This contrast suggests that, while the app may require some initial familiarization, it does not represent a significant barrier to effective use. Overall, the questionnaire results confirm that the application is reliable, easy to use, and suitable for users without advanced technical experience.

### 4.3 Discussion

The results obtained during the validation process demonstrate that the proposed mobile application successfully fulfills its main objective: enabling the detection of diseases in apple tree leaves through images taken or uploaded by the user. The overall success rate of the test cases was 95%, which reflects the correct implementation of the system’s various components, from the user interface to the classification model’s processing.

A particularly notable result was the performance of the test case related to successful detection from clear images, which achieved an 80% success rate in the second test iteration. In the first iteration, this rate was only 40%, revealing initial limitations in the model’s disease detection capabilities. In response, two additional classes were incorporated into the training dataset: Powdery mildew, a common foliar disease, and spider mite, a widespread pest in apple crops in Peru, based on SENASA records and recommendations from one of the interviewed users (Macedonio). Additionally, a pre-filter based on the OpenAI API was integrated to ensure that uploaded images actually depicted plant leaves. These improvements significantly optimized the App2 model, notably enhancing its usability in real-world scenarios.

From the user experience perspective, the questionnaire results indicate positive acceptance of the application. Participants found the app easy to use, clear in presenting results, and well-suited to its intended purpose. Moreover, all users expressed their willingness to use the application again, reinforcing its practical potential in agricultural settings. Although some responses noted a need for initial learning, this did not negatively impact the overall perception of the system’s usability.

## 5 Conclusions and future work

The results obtained throughout the development and validation of the mobile application demonstrate that the proposed solution is effective for detecting diseases in apple tree leaves. The integration of the hybrid model into an accessible mobile environment allowed users to obtain fast and understandable diagnoses from images taken or uploaded directly from their devices. With an overall success rate of 95% in the test cases and positive user feedback, it can be concluded that the application also shows high potential for adoption in real agricultural contexts.

The improvement in model performance, from 40 to 80% in the case of detection using clear images, highlights the importance of adapting the model to real-field conditions. The inclusion of additional classes such as Powdery mildew (a disease) and spider mite (a pest), both commonly found in Peru, enhanced the model’s ability to accurately recognize relevant visual symptoms. The inclusion of a filter to validate the presence of leaves in images was also a key improvement, as it avoided unnecessary processing and reduced detection errors. Together, these technical decisions strengthened the application’s practical utility.

For future work, it is proposed to expand the number of model classes to include more diseases and pests relevant to apple cultivation, particularly those prevalent in the Peruvian context. Additionally, a version with offline capabilities is suggested, enabling the application to function without an internet connection and thus making it more accessible in rural areas. It is also recommended to integrate a geolocation feature that records the location of the crop at the time of image capture, which would allow for the generation of spatial information useful for traceability, incidence mapping, and future early warning systems.

## Data Availability

The original contributions presented in the study are included in the article/Supplementary material, further inquiries can be directed to the corresponding author.
