# Supplementary material for: App2: software solution for apple leaf disease detection based on deep learning (CNN+SVM)
Source: Front Artif Intell. 2025 Oct 15;8:1648867. doi: 10.3389/frai.2025.1648867 (PMC12568702; doi:10.3389/frai.2025.1648867)
Supplement: Supplementary file 1 [file Table_1.DOCX]

Appendix

# Experimental protocol to evaluate the mobile application App2

The objective of this protocol is to validate both the functionality and reliability of the mobile application developed for the recognition of diseases in apple tree leaves, as well as to gather user feedback regarding its usability and practical usefulness. The working hypothesis is the development of a software solution capable of detecting diseases in apple leaves by processing real images captured from mobile devices.

The target audience consists primarily of male farmers aged 36 and above, with a high concentration of elderly individuals (60+ years). They work full-time in agricultural fields, generally from early morning until early afternoon, and reside in rural areas. Their educational background typically ranges from primary to secondary education, but they possess sufficient practical skills to operate mid-range smartphones.

A group of farmers will execute the test cases. The evaluation will be considered satisfactory if at least 80% of the test cases are successfully passed. If necessary, the validation period may be extended by an additional week. Simultaneously, a satisfaction questionnaire will be administered to assess aspects such as the comprehensibility of the model’s output, ease of navigation, and perceived usefulness.

## Materials

Software:

- Google Colab
- OpenAI API
- App2 mobile application
- Defined test cases
- User experience questionnaire
- Result analysis tools (Excel)

Hardware:

- Mobile devices (Android) with a functional camera and internet access

Other materials:

- Internet connection (384 kbps minimum)
- Test logs

## Methods

### Experimental design

The test cases will be executed by a group of five farmers. Each participant will test key features of the mobile app in different usage scenarios, such as uploading an image from a gallery, taking a direct photo, successfully uploading it to the server, and viewing the model's output. A supervisor will verify the process to ensure compliance with the protocol.

**Table A1.** Participants

| **Nombre** | **Rol** |
| --- | --- |
| Erick | Supervisor |
| Macedonio | Farmer |
| Alex | Farmer |
| Yuvan | Farmer |
| Manuel | Farmer |
| Carlos | Farmer |
| Cesar | Support User |
| Jefferson | Support User |

### User experience questionnaire

Users will use the app and answer a subsequent questionnaire. This questionnaire will include Likert-type scale statements (1 to 5). The statements are as follows:

- I think I would like to use this app frequently to detect diseases on apple tree leaves.
- I found the application easy to use.
- I think I would need technical help to be able to use this app.
- The app is well organized and everything makes sense to each other.
- I did notice that sometimes the app would not work the same or would crash.
- Learning to use this app was quick and easy for me.
- I found the app difficult or confusing to use.
- I needed to learn many things before I could use the application correctly.
- The results provided by the model matched your expectations or knowledge about leaf disease.
- The app detected the disease in a reasonable time.

### Step-by-step procedure

**Table A2.** Test Cases

| **ID** | **Description** | **Expected result** |
| --- | --- | --- |
| TC001 | Taking a photo for analysis | The mobile app sends the image to the server for analysis. |
| TC002 | Loading a saved photo | The mobile app sends the image to the server for analysis. |
| TC003 | Successful detection with clear image | The model identifies the disease present on the leaf. |
| TC004 | Display model result | The mobile app displays a section about the model's detection results. |
| TC005 | Diagnostic recommendations | The mobile app displays a section with the recommendations sent from the server. |
| TC006 | Successful access to history | The mobile app quickly loads the history, including all uploaded images, along with their respective dates and diagnoses. |
| TC007 | Deleting a diagnostic history record | The mobile app deletes the record from the user's diagnosis history and from the system. |
| TC008 | Cancel deletion of a diagnostic history record | The system does not delete the record. |
| TC009 | Successful registration with basic data | The system adds a new user to the database. |
| TC010 | Weak or mismatched password | The mobile app displays a warning that the password is weak or does not match, prompting the user to try again. |
| TC011 | Successful login | The mobile app displays the user's session. |
| TC012 | Failed login | The mobile app displays a message about incorrect credentials. |
| TC013 | Update user data | The system updates the user's data. |
| TC014 | Account deletion | The system permanently deletes all data associated with the user's account, including images, diagnoses, settings, and any other saved information. |
| TC015 | Cancel account deletion | The system does not delete the user's account. |
| TC016 | Update notification | The mobile app displays details of each update. |
| TC017 | Show guidance | The mobile app displays a section that explains step-by-step how the app works. |

**Detailed instructions:**

- Each user will install the app on their device.
- Each case will be documented.
- Selected users will use the app on their own and complete the questionnaire.

### Controls

- Field tests will be conducted where there is an internet connection (384 kbps minimum).

### Repetitions and number of samples

The test cases were carried out in the Huaura district of the Huaura province, located in the Lima department in Peru, georeferenced: <https://maps.app.goo.gl/mg6BmhKRU7z5Wxve6> conducted during weeks 7 and 9 of the work schedule, from May 13 to May 30, 2025. Each test case will be executed twice (once by each user) to verify consistency. Approximately 17 unique test cases will be evaluated, resulting in 34 individual executions. Each participating user will complete the questionnaire after testing the application.
